# Prospective cohort study of regenerative potential of non vital immature permanent maxillary central incisors using platelet rich fibrin scaffold

**DOI:** 10.1038/s41598-021-93236-2

**Published:** 2021-07-01

**Authors:** Selvakumar Kritika, V. Sujatha, N. Srinivasan, Senthil Kumar Renganathan, Sekar Mahalaxmi

**Affiliations:** 1 Department of Conservative Dentistry and Endodontics, SRM Dental College, SRM Institute of Science and Technology, Tamil Nadu, Ramapuram Campus, Bharathi Salai, Ramapuram, Chennai, 600 089 India; 2 Specialist Endodontist, Hamad Dental Center, Hamad Medical Corporation, Doha, Qatar; 3 Department of Conservative Dentistry and Endodontics, Rajas Dental College and Hospital, Kavalkinaru Junction, Tirunelveli, Tamil Nadu 627105 India

**Keywords:** Cell biology, Stem cells, Health care, Medical research

## Abstract

Regenerative endodontic procedures have gained momentum as a treatment modality of young immature permanent teeth. Literature reports reveal that platelet-rich fibrin (PRF) stimulates growth factors and induces regeneration. This study was undertaken to assess the regenerative potential of non-vital immature permanent maxillary central incisors using PRF with a follow-up for 2 yrs. 19 patients in the age group of 9–25 yrs with immature, non-vital permanent maxillary central incisors (n = 23) with/without signs and/or symptoms of periapical pathosis and open apex were included in this study. In the first appointment, access opening, canal disinfection and triple antibiotic paste placement were done. In the subsequent visit, PRF was prepared and placed inside the canal. Access was sealed with Mineral trioxide aggregate plug and composite. The patient was reviewed up to 24 mths. The mean difference was statistically analyzed using Friedman test followed by Dunn post hoc test and adjusted by Bonferroni correction (*p* < 0.05). As per AAE guidelines, the primary and secondary goals were achieved. A significant (*p* < 0.001) gradual increase in the root length, thickness of dentinal walls and decrease in apical diameter were observed. Within the limitations of this study, PRF placement was clinically and radiographically effective in inducing regeneration of non-vital immature permanent teeth.

## Introduction

Pulp necrosis as a result of trauma or other insults interrupts the process of root maturation and apical closure in young permanent teeth^1^. A twelve-year review of literature states that 25% of school children and 33% of adults experience trauma to their permanent dentition with the majority of these occurring before the age of 19 years^2^. The treatment of young immature permanent teeth with pulp necrosis is therefore challenging to dental practitioners^3^. A paradigm shift towards regenerative endodontics procedures (REP) facilitated continued root maturation and apical closure. REP is defined “as a biological procedure that predictably replaces damaged, diseased or missing structures including dentin and root structures as well as cells of the pulp-dentin complex (PDC), with viable tissues preferably of the same origin, that restore the normal physiologic functions of the PDC”^4^.

Tissue engineering and tissue regeneration form the backbone of REP; the former utilizes specific stem cells, three-dimensional scaffolds and growth factors to regenerate PDC, the latter involves disinfection and revascularization of the PDC^4,5^. do Couto et al. stated that the disinfection of the immature teeth in REP can be judiciously achieved with the use of triple antibiotic paste (TAP)^6^. Although the current clinical recommendations by the American Association of Endodontists (AAE) for REP suggests the use of TAP or calcium hydroxide, when comparatively analysing, TAP and double antibiotic paste showed increased antimicrobial activity compared to calcium hydroxide^7^.

A scaffold is a physicochemical three-dimensional structure facilitating cellular organization and vascularisation. Natural scaffolds such as platelet-rich plasma (PRP) and platelet-rich fibrin (PRF) are advantageous over synthetic polymers and collagen in terms of cost, inflammatory reactions, immune responses and cytotoxicity^8^.

According to the currently available data, revascularization through blood clot technique and scaffold implantation remains the techniques that have been clinically attempted. Although revascularization using a blood clot was effective, the clot formed was unpredictable to serve as a scaffold. On the other hand, scaffold implantation with platelet concentrates delivers an enhanced concoction of growth factors and offers mechanical sustenance^9^. Visser et al. reported that the platelet concentrates have increased concentration of growth factors and increased cellular proliferation when compared to the blood clot^10^.

Platelet-rich fibrin is a second-generation platelet concentrate. Ehrenfest et al. classified platelet concentrate into four main categories namely, pure PRP (P-PRP), leukocyte-rich PRP (L-PRP), pure PRF (P-PRF) and leucocyte-rich PRF(L-PRF). The leucocyte-rich PRF includes the Choukron’s PRF, advanced PRF (A-PRF) and injectable PRF (I-PRF) ^11^. PRF is an autologous tri-molecular or equilateral fibrin branched junctional matrix which is expedient over PRP in terms of ease of preparation and use of anticoagulants^12,13^. According to Huang et al., PRF can stimulate pulp cell proliferation, enhance osteoprotegrin protein expression and induce alkaline phosphatase activity^5^.

According to data analysis on clinical protocols in REP, the overall percentage of studies in which intracanal bleeding or PRP/PRF was used is only 13% with insufficient evidence using a large sample size available in the literature^14^.

Hence the aim of this in vivo prospective cohort study was to evaluate the regenerative potential of scaffold implantation technique with PRF in non-vital immature permanent maxillary central incisors with a follow-up period of 2 years. In accordance with the AAE guidelines (8.5.2014), the primary and secondary goals were set as follows:

Primary: Elimination of the symptoms of pain /swelling (before scaffold implantation procedure) with evidence of bony healing and resolution of apical radiolucency (observed 6–12 months after treatment).

Secondary: Increase in root length and root dentin thickness (observed at 12–24 months after treatment) and decrease in apical diameter.

Tertiary: Positive pulp vitality response (which if achieved, could indicate a more organized vital pulp tissue).

## Methods

### Patient recruitment

**Figure 1:**
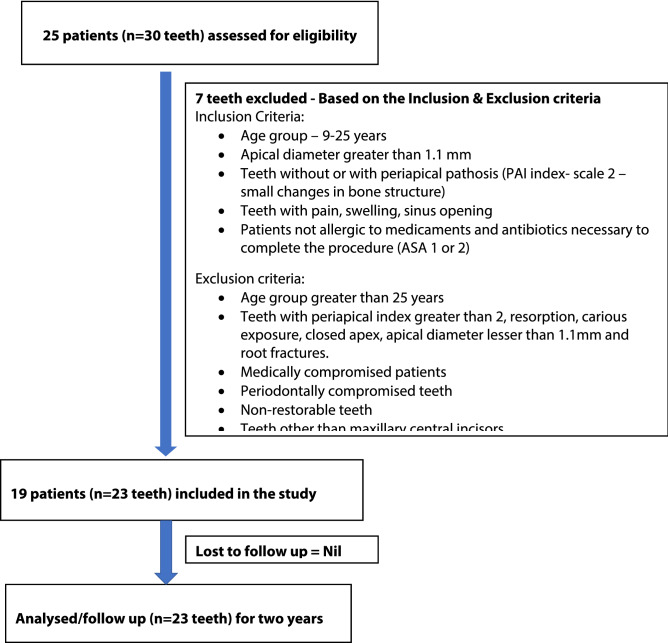
Flowchart according to STROBE guidelines.

Nineteen patients (4 patients with 2 teeth each) in the age group of 9–25 years with immature, non-vital permanent maxillary central incisors (n = 23) with/without signs and/or symptoms of periapical pathosis (PAI index-up to scale 2–small changes in bone structure) and open apex (apical diameter greater than 1.1 mm) were included in this study. The study protocol was presented to the Institutional Review Board and approval was obtained based on the regulation of the Ethical Committee of the Faculty of Dentistry, SRM Institute of Science and Technology (SRMDC/IRB/2014/MDS/No.304) and registered in Clinical Trial Registry of India (CTRI/2017/10/010,246) (30/10/2017). Patients were explained about the treatment protocol and informed consent was obtained from the patient and his/her parent (if the patient was less than 18 years of age). Detailed medical and dental histories, and clinical examination with pulp sensibility testing and radiographic examination of the involved teeth were carried out to confirm the diagnosis. Teeth with closed apex, caries lesions, periodontally compromised teeth, resorption, teeth with root fracture, teeth other than maxillary central incisors were excluded. All procedures were carried out in accordance with the Ethical Committee guidelines of SRM Institute of Science and Technology and AAE guidelines for regenerative endodontics (8.5.2014). The flowchart according to the STROBE guidelines is depicted in Fig. 1.

### Treatment procedure

#### Appointment 1

Local anesthesia (LA) was administered using 2% lignocaine without vasoconstrictor by infiltration technique. Under rubber dam isolation and aseptic conditions, the access cavity was prepared using sterile No:1 Endo access and Endo ‘Z’ bur (Dentsply Maillefer, USA). After working length (WL) determination, minimal instrumentation was done and the canal was irrigated using 10 ml of 1% sodium hypochlorite (NaOCl) (Chenchems Reagent, Chenchemicals, Chennai, India) for 2 min with side vented needles placed 1 mm short of WL. The canals were dried using paper points 2 mm short of the WL. 0.02 mg each of ciprofloxacin, metronidazole and amoxicillin was mixed in the ratio of 1:1:1 with 0.5 mL of propylene glycol to obtain a final concentration of 0.12 mg/mL which was placed in the coronal third of the root canal. The pulp chamber was then temporarily restored with Cavit G (3 M ESPE, Germany) and a radiograph was taken. The patient was recalled after 3 weeks.

#### Appointment 2

##### Preparation of PRF

10 mL of whole blood was withdrawn from the median cubital vein of the patient using 10 mL disposable syringe and needle. The blood was collected in a sterile glass test tube and centrifuged (REMI centrifuge, Mumbai, India) at 3300 rpm for 12 min at 400 g force. The resultant membrane was then placed between 2 sterile gauze pieces and squeezed to form a strong fibrin network.

##### Placement of PRF

3% mepivacaine without vasoconstrictor (Scanodest, Septodont, Lancaster, UK) was administered. Under aseptic conditions and rubber dam isolation, the temporary restoration was removed and the TAP was flushed out copiously with saline. Canal irrigation was done using 10 mL of 1% NaOCl and a final rinse with 5 mL of 17% EDTA (Chenchems Reagent, Chenchemicals, Chennai, India) solution. The canal was dried with paper points and the thin strands of PRF were cut and placed into the root canal space using Buchanan hand pluggers (Sybron Endo, Glendora, CA) 1 mm beyond the working length and coronally just below the CEJ. MTA (MTA Angelus, Angelus, Brazil) plug of 3 mm was packed coronally over the PRF scaffold. The canal orifice was sealed with Type II glass ionomer cement (GC, Tokyo, Japan) and the tooth was finally restored using composite resin (Te-Econom Plus, Ivoclar Vivadent, Mumbai, India) and a digital radiograph was taken.

### Follow-up analysis

The routine follow-up was carried out using clinical and radiographic assessments at 3, 6, 9, 12, 15, 18-month intervals and for a period of 24 months. The standardization of radiographic assessment was done using a radiographic platform (RINN XCP-DS alignment system Gendex Visualix, Dentsply, UK). The radiographs were taken under paralleling technique. The radiographs were digitally recorded and quantitative analysis was done using the SOPRO imaging software (Supplementary Fig. S1). As stated earlier, the primary and secondary objectives were measured in the follow-up analysis. Two independent evaluators who were not part of the treatment protocol performed the measurements and the mean ± standard deviation was taken and analyzed. Since the coronal thirds of all the teeth were fully developed, we analyzed the changes in the dimensions of the middle and apical thirds of the mesial and distal dentinal walls. Based on Chen et al. classification, the type of responses in the regeneration of immature teeth was assessed in the 23 cases as follows : type I—increased thickening of the canal walls and continued root maturation, type II- no significant continuation of root development with blunt and closed root apex, type III- continued root development with open apical foramen, type IV- severe calcification (obliteration) of the canal space and type V- formation of a hard tissue barrier between the coronal MTA plug and the root apex^15^. Under rubber dam isolation, pulp sensibility assessment was done by cold test using ethyl-chloride spray (Roeko Endo-Frost, COLTENE, USA) and heat test using hot water at each time interval.

### Statistical analysis

The quantitative measurements obtained from radiographs of different time periods were tabulated in an excel sheet. The mean increase in root length, mean increase in thickness of dentinal wall and the mean decrease in the apical diameter values were obtained. For all patients, the preop values of all 3 parameters were set at zero for standardisation and only the increase or decrease in values (in mm) which indicates the regenerative potential was quantitatively analysed over the follow up periods (i.e. postop- preop). These values were analyzed statistically using SPSS software version 22. The data were assessed for normality and found to be non-normal in distribution. Hence non-parametric test, the Friedman test followed by the Dunn post hoc test with Bonferroni correction was employed to detect the statistically significant difference in the regeneration (assessed by the various parameters—apical diameter, root length and thickness of dentin wall) over a period of 24 months. The p-value was set at 5%.

## Results

The primary objective was achieved in all the patients, wherein no patients reported symptoms, clinical and radiographic signs of failure during any of the follow-up periods. Out of the 23 cases, 7 cases had a periapical lesion. At 6 months follow-up period, in 5 cases complete resolution of symptoms and periapical radiolucency’s with complete bone healing was observed and in 2 cases substantial evidence of bony healing was noted. Therefore complete periapical bone healing was evidenced in 90% of the cases. The mean and standard deviation of various parameters assessing the regeneration is mentioned in Table 1. A significant increase in root length, the thickness of the dentinal wall in the apical third of the root dentin was observed from 12 months onwards when compared to the preop values (*p* < 0.001). However, the mesial and distal dentin thickness at the middle third showed a statistically significant increase only from 18 and 24 months onwards (*p* < 0.05). A highly significant decrease in apical diameter was noticed from 12 months onwards (*p* < 0.001).

**Table 1:** Mean (Standard deviation) of difference between Pre- and Post-operative values of various parameters.

Parameters	Pre-Op	3 Months	6 Months	9 Months	12 Months	15 Months	18 Months	24 Months
Apical Diameter (mm)	0^a^	0 ^a^	0^a^	0.300^a^ (0.47)	**0.83**^**b**^** (0.38)**	**1.09**^**b**^** (0.288)**	**1.35**^**b**^** (0.487)**	**1.83**^**b**^** (0.388)**
Root Length (mm)	0 ^a^	0^a^	0^a^	0.48^a^ (0.51)	**0.91**^**b**^** (0.28)**	**1.17**^**b**^** (0.388)**	**1.57**^**b**^** (0.507)**	**1.91**^**b**^** (0.417)**
Thickness of Mesial Dentinal wall- Apical third (mm)	0 ^a^	0^a^	0^a^	0.04^a^ (0.20)	**0.70**^**b**^** (0.47)**	**0.96**^**b**^** (0.209)**	**1.00**^**b**^** (0.000)**	**1.04**^**b**^** (0.209)**
Thickness of Distal Dentinal wall – Apical third (mm)	0 ^a^	0 ^a^	0^a^	0.43^a^ (0.50)	**0.87**^**b**^** (0.34)**	**1.00**^**b**^** (0.000)**	**1.04**^**b**^** (0.209)**	**1.04**^**b**^** (0.209)**
Thickness of Mesial Dentinal wall- Middle third (mm)	0^a^	0^a^	0^a^	0^a^	0.13^a^ (0.34)	0.30^a^ (0.470)	**0.48**^**b**^** (0.511)**	**0.57**^**b**^** (0.507)**
Thickness of Distal Dentinal wall- Middle third (mm)	0^a^	0^a^	0^a^	0^a^	0^a^	0.26^a^ (0.449)	0.30^a^ (0.470)	**0.52**^**b**^** (0.511)**

^a,b^Different alphabetical superscript for each time period indicates the statistical significant difference (*p* < 0.05).

## Discussion

The prerequisites essential for root maturation include the presence of wide-open apex for tissue ingrowth, young aged individuals where high stem cell regeneration potential is present, cautious use of sodium hypochlorite as an irrigant for effective disinfection and the use of triple antibiotic paste as intracanal medicament^3^. Another major factor is the initiation and/or placement of a scaffold that creates a platform for regeneration to occur. An appropriate scaffolding must yield a correct spatial location of stem cells and regulate their differentiation, proliferation and metabolism by inducing growth factors^12^. The growth factors present in PRP and PRF such as platelet-derived growth factor, transforming growth factor, insulin-like growth factor, etc. They stimulate mitogenic response leading to proliferation and differentiation of the Stem Cells of Apical Papilla (SCAP) into odontoblasts which are influenced by the cells of the Hertwig’s epithelial root sheath (HERS)^4^. These odontoblasts deposit atubular dentin at the apical and lateral walls of the root canal leading to continued root maturation^16^.

Simonpieri et al. stated that PRF acts as a biological connector between bone particles thereby maintaining and protecting grafted biomaterials, facilitates cell migration for neoangiogenesis, promotes healing by the release of cytokines, upregulates the inflammatory process and acts as an anti-infectious agent^17^. Ulusoy et al. compared the use of a blood clot, PRP, PRF and platelet pellets as scaffolds for REP and concluded that the platelet derivatives yielded a higher rate and prolonged exposure to the growth factors with fewer chances of obliteration of the canal space^18^.

Studies have proven the effectiveness of revascularization procedures in the adolescent age groups because of an increase in the differentiating ability of the stem cells in terms of quantity, quality and mobilization capacity unlike in aged individuals. Therefore a wide range of patients between the age group of 9 to 25 years was chosen in the study. Avoiding mechanical debridement of canal walls and use of lower concentration of NaOCl further ensures the vitality of SCAP^19^. NaOCl at concentrations greater than 3%is found to be cytotoxic to periodontal ligament cells and SCAP cells^20^. Baumgartner et al. and Siqueira et al. proved that 1%NaOCl provided adequate tissue dissolving and antibacterial properties^21,22^. Martin et al. stated that 17%EDTA when used as the final irrigant partially, reverses the detrimental effects of high concentrations of sodium hypochlorite solution and has a positive effect on the survival and differentiation of SCAP^23^. Generally, immature teeth are more difficult to disinfect with antimicrobials regardless of the concentration of NaOCl used^24^. Hence the use of intracanal medicament in the form of TAP enables better disinfection. Devaraj et al., proved that TAP completely disrupts the biofilm, unlike DAP where the biofilm is structurally altered and not completely eradicated^25^. The modified version of TAP suggested by Thomson et al., where minocycline was replaced with amoxicillin and used in combination with ciprofloxacin and metronidazole to prevent discoloration was employed in our study^26^. An optimal coronal seal is essential to prevent the ingress of bacteria. Therefore a tight coronal seal was established with 3–4 mm thickness of MTA placed coronally over the PRF scaffold^27^.

The primary objective of REP was achieved at 6 months follow-up period, in all the cases with complete resolution of symptoms and periapical radiolucency’s, where existed. Literature reveals few studies on quantitative analysis in REP^28–30^. Hence our study also focussed on the quantitative radiographic assessment of the secondary objectives such as root length, apical diameter and thickness of dentinal walls. A significant overall mean decrease of 88.40%in apical diameter and an increase of 16.92%in root length were observed. There was a significant increase in the thickness of both the walls in the middle and apical thirds. When compared with the preoperative values, a significant difference in all the parameters was observed from the 12th month onwards.

**Figure 2:**
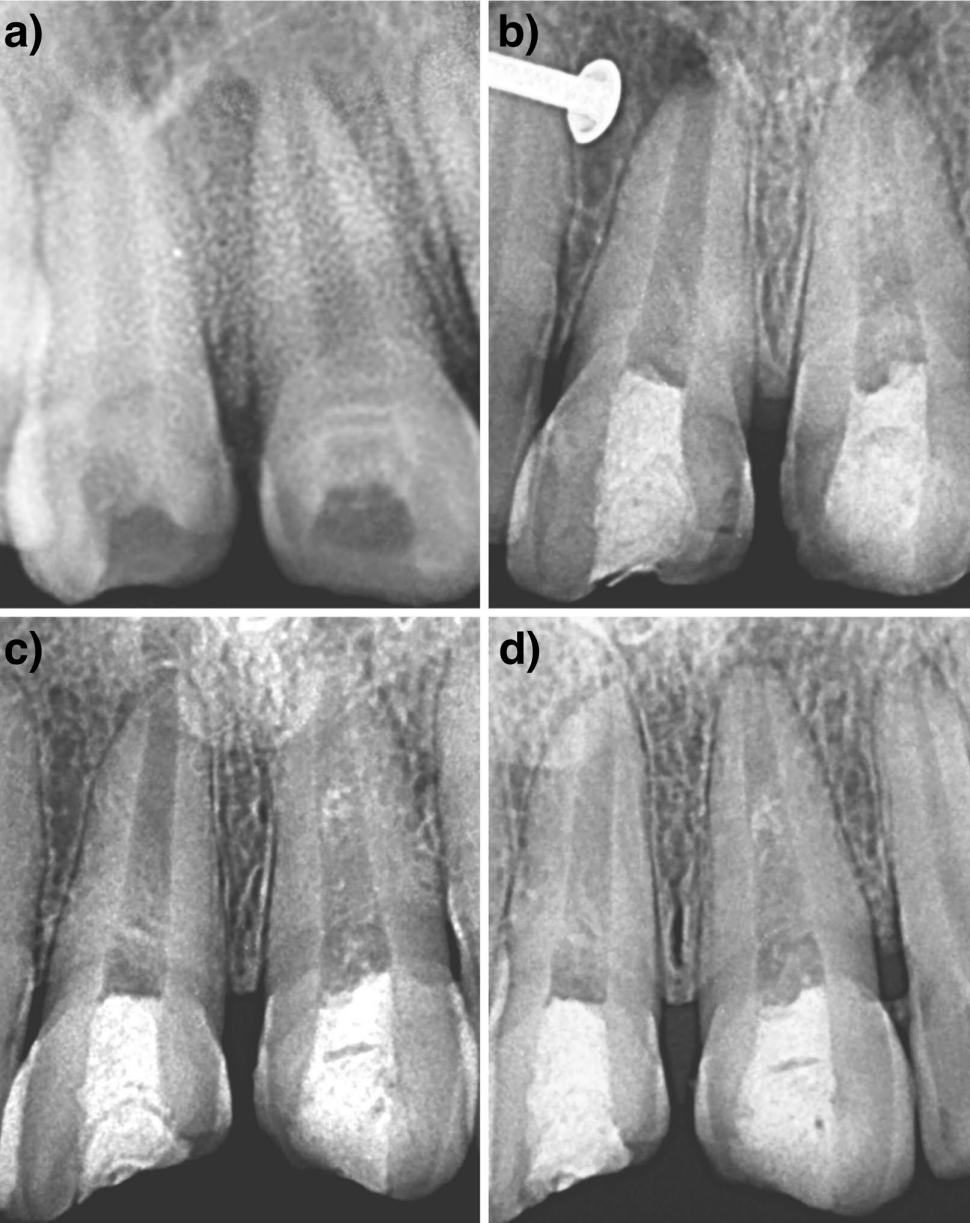
Case 1: Tooth no. 11, 21- (**A**) Preoperative radiograph; (**B**–**D**) Radiographs at 6 months, 12 months and 24 months follow-up period.

**Figure 3:**
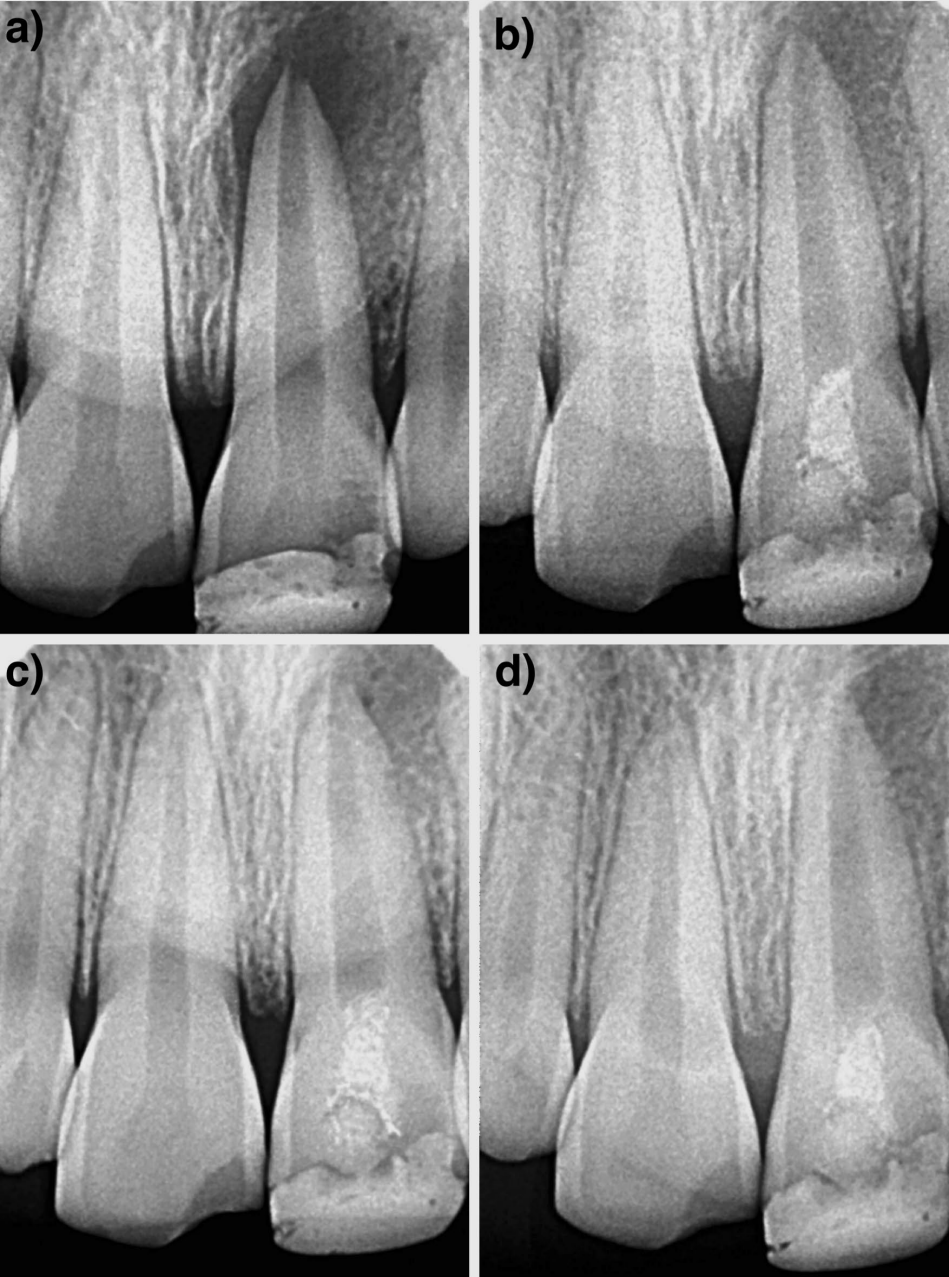
Case 2: Tooth no. 21- (**A**) Preoperative radiograph; (**B**–**D**) Radiographs at 6 months, 12 months and 24 months follow-up period.

**Figure 4:**
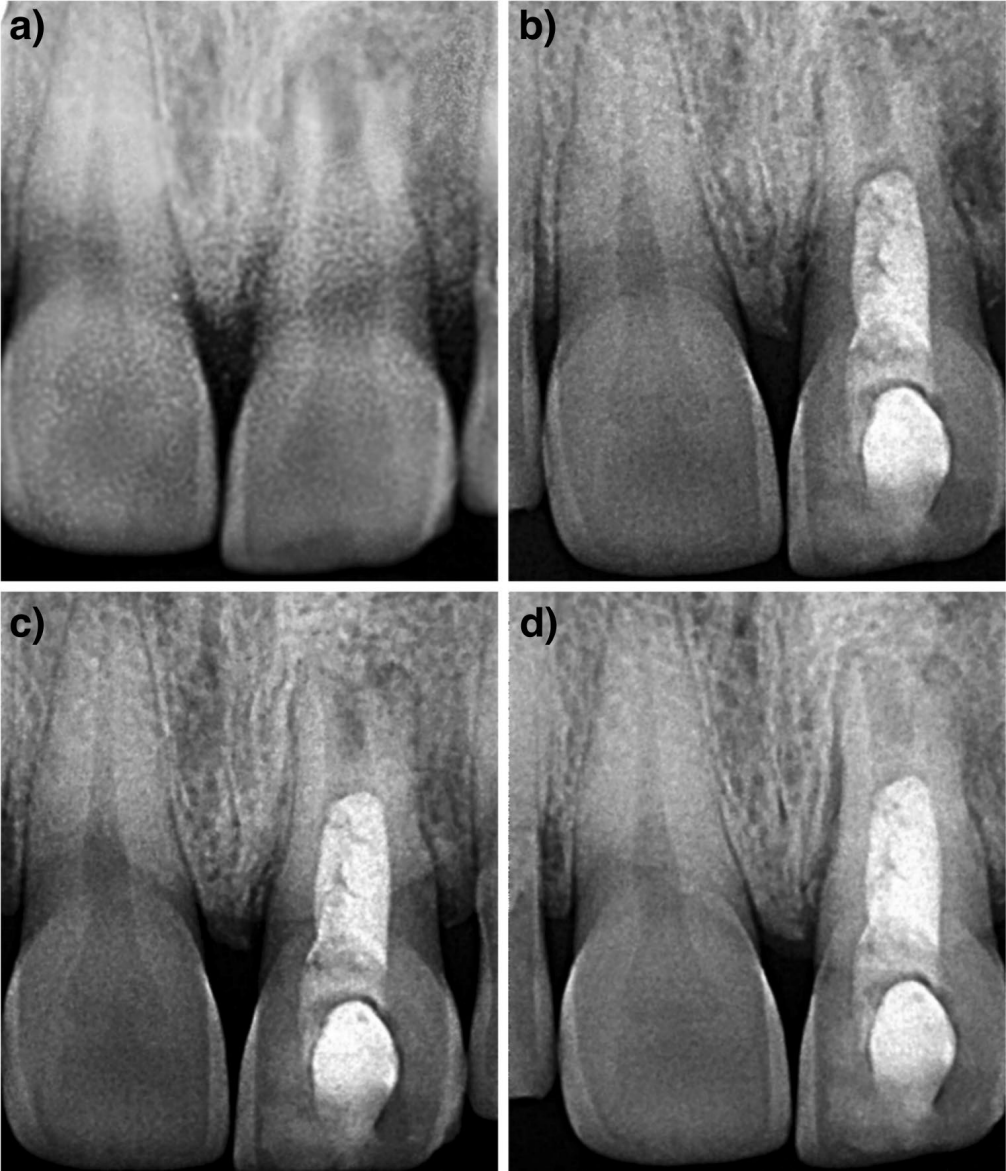
Case 3: Tooth no. 21- (**A**) Preoperative radiograph; (**B–D**) Radiographs at 6 months, 12 months and 24 months follow-up period.

In our study at 2 years, Type III was observed in all the teeth except one, suggesting complete root maturation (Figs. 2 and 3). In only one case, a type V response was noticed (Fig. 4). The probable reason could have been the MTA placement up to the middle third which induced the hard tissue barrier formation.

Based on the literature evidence put forth the following mechanisms for root maturogenesis can be considered:

- Few vital pulp cells remain at the apical canal end which has the ability to resist destruction even in the presence of inflammation and can proliferate and differentiate into odontoblasts^27^.
- The periodontal ligament stem cells can proliferate and grow within the apical end of the canal lumen through the open apex in cases where there is the destruction of HERS and SCAP^31,32^.
- Instrumentation and placement of PRF beyond the apical foramen initiates the transplantation of SCAP into the canal lumen, wherein the SCAP are resistant to infection and retain their capability to undergo proliferation and differentiation into odontoblasts and osteoblasts^33,34^.
- The PRF acts as a reservoir of growth factors that stimulate the differentiation, growth and maturation of fibroblasts, odontoblasts and cementoblasts from their undifferentiated precursors^35^.

Although controversies exist on the clinical vitality status of the teeth treated with REP, the pulp sensibility assessment—cold test carried out during the follow-up period was found to be negative, probably due to the thick coronal seal of 3-4 mm with MTA and composite resin diminishing the chances to know the exact vitality status.

According to Ritter et al., in the study conducted on dog teeth, it was reported that the ingrowth of tissue is more likely to originate from periodontal ligament consisting of bone, cementum and dentin-like material rather than pulp tissue^36^. As there is limited literature evidence substantiating the type and nature of newly formed tissue, further studies have to be undertaken in this aspect.

Limitations:

1. The software used in the present study could measure only the mesio-distal aspect of the teeth. A 3-dimensional assessment can possibly be carried out with the use of advanced technological aids like CBCT.
2. Grey MTA containing bismuth oxide used in the study resulted in discoloration of all teeth. The placement of a 3 mm MTA plug in the coronal third of the root canal would have caused the discoloration of the teeth. This could be possibly avoided by the use of Biodentine (Septodont, Lancaster, UK).

Future studies can be undertaken towards the use of newer generation leucocyte-rich PRF scaffolds for REP such injectable PRF (I-PRF). I-PRF can be injected into the application site and form a small clot a few minutes after the injection. Besides, it contains higher concentrations of platelets, leukocytes, stem cells and growth factors^37^.

## Conclusion

Within the limitations of the present in vivo prospective cohort study, it can be concluded that the use of platelet-rich fibrin as a scaffold has proven to be clinically and radiographically effective in terms of increase in root length, decrease in apical diameter and increase in dentin wall thickness in all subjects when tested over a 2 year follow up period. Thus regenerative endodontics using scaffold implantation technique (PRF) can be a viable alternative treatment option over other techniques for non-vital immature permanent teeth.

## Supplementary Information

Supplementary Information.

## Data Availability

All data generated or analysed during this study are included in this published article (and its Supplementary Information files).
