# Supplementary material for: Prospective cohort study of regenerative potential of non vital immature permanent maxillary central incisors using platelet rich fibrin scaffold
Source: Sci Rep. 2021 Jul 1;11:13679. doi: 10.1038/s41598-021-93236-2 (PMC8249665; doi:10.1038/s41598-021-93236-2)
Supplement: Supplementary file 1 — Supplementary Information. [file 41598_2021_93236_MOESM1_ESM.pdf]

Prospective Cohort Study of Regenerative Potential of Non Vital Immature Permanent Maxillary Central Incisors using Platelet Rich Fibrin Scaffold

Selvakumar Kritika[1*], V Sujatha[1], N Srinivasan[2], Senthil Kumar Renganathan[3], Sekar Mahalaxmi[1]

[1]Department of Conservative Dentistry and Endodontics, SRM Dental College, Ramapuram, SRM Institute of Science & Technology, Ramapuram Campus, Bharathi Salai, Ramapuram, Chennai 600 089, Tamil Nadu, India.

[2]Specialist Endodontist, Hamad Dental Center, Hamad Medical Corporation, Qatar

[3]Department of Conservative Dentistry and Endodontics, Rajas Dental College & Hospital, Kavalkinaru Junction, Tirunelveli 627105, Tamil Nadu, India

*Corresponding author: Phone No: +91-44-22492882, Fax No: +91-44-22491777; Mobile No: +91-8072197199; E-mail address: drkritikaselvakumar22@gmail.com

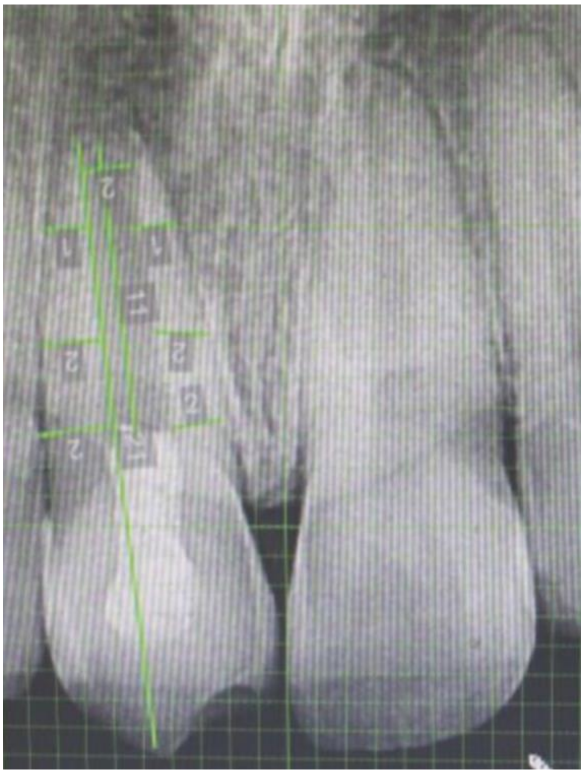

Fig. S1: Representative image depicting the quantitative measurements of apical diameter, root length and thickness of dentine wall at coronal, middle and apical third using SOPRO imaging software.
